# Efficient Light Management in a Monolithic Tandem Perovskite/Silicon Solar Cell by Using a Hybrid Metasurface

**DOI:** 10.3390/nano9050791

**Published:** 2019-05-23

**Authors:** Mahmoud H. Elshorbagy, Braulio García-Cámara, Eduardo López-Fraguas, Ricardo Vergaz

**Affiliations:** 1 GDAF-UC3M, Departamento de Tecnología Electrónica, Universidad Carlos III de Madrid. Avda. Universidad, 30. Leganés, E28911 Madrid, Spain; melshorb@pa.uc3m.es (M.H.E.); edlopezf@ing.uc3m.es (E.L.-F.); rvergaz@ing.uc3m.es (R.V.); 2 Grupo Complutense de Óptica Aplicada, Departamento de Óptica, Facultad de Óptica y Optometría, Universidad Complutense de Madrid, E28037 Madrid, Spain

**Keywords:** metasurface, perovskite solar cell, monolithic tandem solar cell, short-circuit current, numerical optimization

## Abstract

Solar energy is now dealing with the challenge of overcoming the Shockley–Queisser limit of single bandgap solar cells. Multilayer solar cells are a promising solution as the so-called third generation of solar cells. The combination of materials with different bandgap energies in multijunction cells enables power conversion efficiencies up to 30% at reasonable costs. However, interfaces between different layers are critical due to optical losses. In this work, we propose a hybrid metasurface in a monolithic perovskite-silicon solar cell. The design takes advantage of light management to optimize the absorption in the perovskite, as well as an efficient light guiding towards the silicon subcell. Furthermore, we have also included the effect of a textured back contact. The optimum proposal provides an enhancement of the matched short-circuit current density of a 20.5% respect to the used planar reference.

## 1. Introduction

Silicon solar cells are still in a dominant position in the photovoltaics market. Therefore, new strategies should be taken into account to improve their performance if we want to push solar energy up [1]. New technologies have arisen to overcome the main efficiency limitation: the Shockley–Queisser limit [2]. The Shockley–Queisser limit establishes the maximum efficiency of a single junction solar cell 30% at a band gap of 1.1 eV [2]. This calculation used a simplified model of the solar spectrum, while more recent calculations give a maximum efficiency of 33.7% at an optimized band gap of 1.34 eV [3]. This means that, having an ideal design of a single junction solar cell based on a material with a band gap of 1.34 eV, under the standard solar spectrum illumination (AM1.5G, 1000 W/m^2^), only 33.7% of that power can be turned into electricity (337 W/m^2^). However, the most popular material used in the market up to now has been silicon, which has a less favorable band gap of 1.1 eV, so the maximum theoretical efficiency is reduced to about 32%. Recent commercial mono-crystalline solar cells have a 24% conversion efficiency, but they can even achieve up to 26% in laboratory conditions [1]. The difference between the efficiency of practical devices and their theoretical limit mainly comes from reflective, parasitic and resistive losses through the different interfaces of the device and reducing some of them is our concern in this work.

A stack of two or more junctions from materials with different band gaps is able to overcome the Shockley–Queisser limit of the single p-n junction up to a very high efficiency value, depending on the used materials, the number of layers and the structural design. The asymptotic limit for a multi-junction solar cell with an infinite number of layers, has a value of 86.8% efficiency, using concentrated sunlight [4].

In the simplest multi-junction device, which is a monolithic tandem solar cell, high-energy photons are absorbed in the top layer of the higher bandgap, while low-energy photons contribute to the photocurrent in the bottom layer. Maximum efficiency is obtained in multi-junction solar cells because they have lower thermalization losses, which is due to the better adaptation in energy levels of the incident photons, leading to a lower loss in carrier extraction potential [5].

The research during the last years shows that lead halide perovskite materials are a good candidate to compose tandem devices with silicon solar cells [6,7]. Their low price, high efficiency [8], sharp optical response and a tunable bandgap near the silicon one (1.5–1.8 eV) make them ideal to combine with current silicon technology. The first monolithic tandem solar cell combining a perovskite and a silicon heterojunction was proposed in 2016 [9], and several research groups are now working on them [10,11].

The way in which the different solar cells compose a multi-junction device is a key issue in order to optimize its final response. A mechanical stack of independent subcells is known as a four-terminal configuration. As the subcells are independently fabricated, their performances can be also independently optimized, avoiding other constraints such as current matching, multilayer deposition compatibility and the creation of tunnel junctions [12]. However, this architecture has important disadvantages. Each subcell has separated top and bottom electrical contacts. Besides, an insulator transparent layer should be introduced in between, and this usually involves remarkable losses. Additionally, the mechanical stacking demands a transparent interlayer medium (e.g., glass) providing a sufficient index matching to avoid high optical losses. In contrast, the monolithic or two-terminal option includes two or more active regions separated by a single tunnel junction in a unique device, in such a way that the fabrication process includes all the considered multi-junctions. This approach is very attractive because it simplifies the electronic circuits and it does not need the insulator intermediate layer previously mentioned, thus simplifying the management of the optical losses. Although this is a promising solution, it requires a current matching between the top and the bottom cell for proper operation, and there are still important technological challenges that should be confronted. The main sources worsening the response of these cells lie on parasitic absorption, non-radiative recombination, undesirable resistances and optical losses [13].

From an optical point of view, there are several innovative strategies to improve the performance of these solar cells, like reducing the reflectivity at interlayers, or increasing the optical path in the active regions. In the first case, the control of the reflectivity in the different layers is mainly focused on the front and bottom layers of the solar cell. The inclusion of selective reflectors [14,15] or the texturization of those layers [16] provide good results, with improvements (*ΔJ_S_*_C_) from 0.82 mA/cm^2^ [17] to 1.3 mA/cm^2^ [18] in the short-circuit current density (*J_SC_*). On the other side, engineered interlayers are considered to increase the light path or to confine light inside the active regions. The inclusion of resonant nanostructures, either plasmonic [19] or dielectric [20,21,22], has been already proposed with remarkable improvements in the light management and consequently in the performance of the device. Recent works have studied the effect of resonant nanostructures embedded in Perovskite solar cells, showing an experimental enhancement of the efficiency of perovskite solar cells over the 6%, being theoretically over the 10% [23,24].

In this work, we propose the design of a monolithic perovskite/silicon tandem solar cell in which the perovskite region is set up as a metasurface. This nanostructured perovskite layer has been designed to improve the optical response of the solar cell by two main effects: an optimum light guiding to the silicon cell through a diffractive effect in the Silicon absorption wavelength range, and a large confinement of short-wavelength light inside the perovskite, increasing its absorption. This metasurface provides also extra grades of freedom: its geometrical parameters. They allow for optimizing the performance of the solar cells by ensuring a current matching between the two component subcells, which is mandatory in a monolithic tandem cell. Additionally, a textured back-contact has been included to increase the performance of our model. We have evaluated the proposal in terms of fabrication, taking account the current state of the art, and it has real potential to produce high-efficiency tandem solar cells without drastic changes in the fabrication process.

## 2. Proposed Structure and Simulation Methods

**Figure 1:**
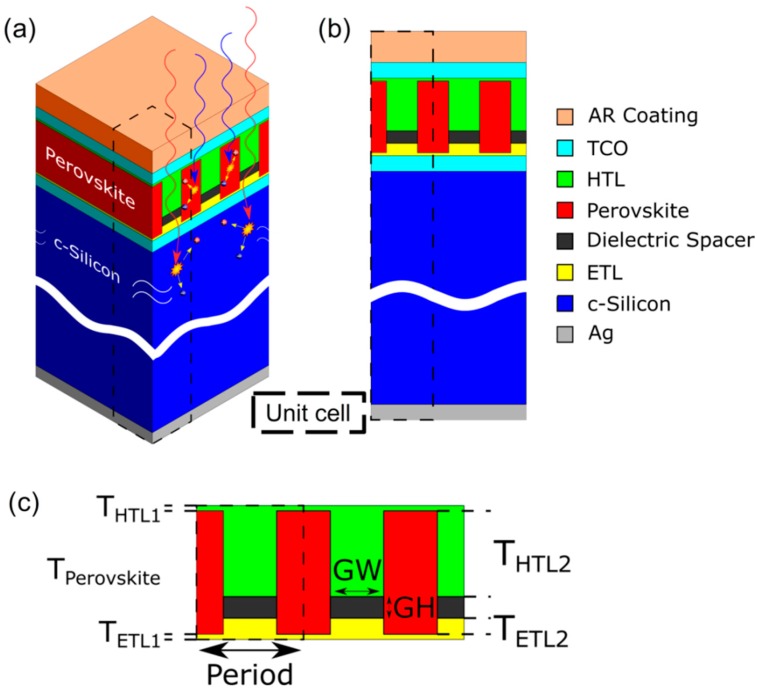
3D (**a**) and 2D (**b**) schemes of the proposed nanostructured configuration of a monolithic perovskite-silicon tandem solar cell. The considered structure allows a guiding effect of photons of high wavelengths (red lines in Figure 1a) towards the silicon layers, while short wavelengths (blue lines) generate electron-hole pairs in the perovskite layer. The detail of the layers and the considered unit cell (dashed boxes) of our simulations are also included. (**c**) Detail of the nanostructure with the definition of the corresponding geometrical parameters.

**Figure 2:**
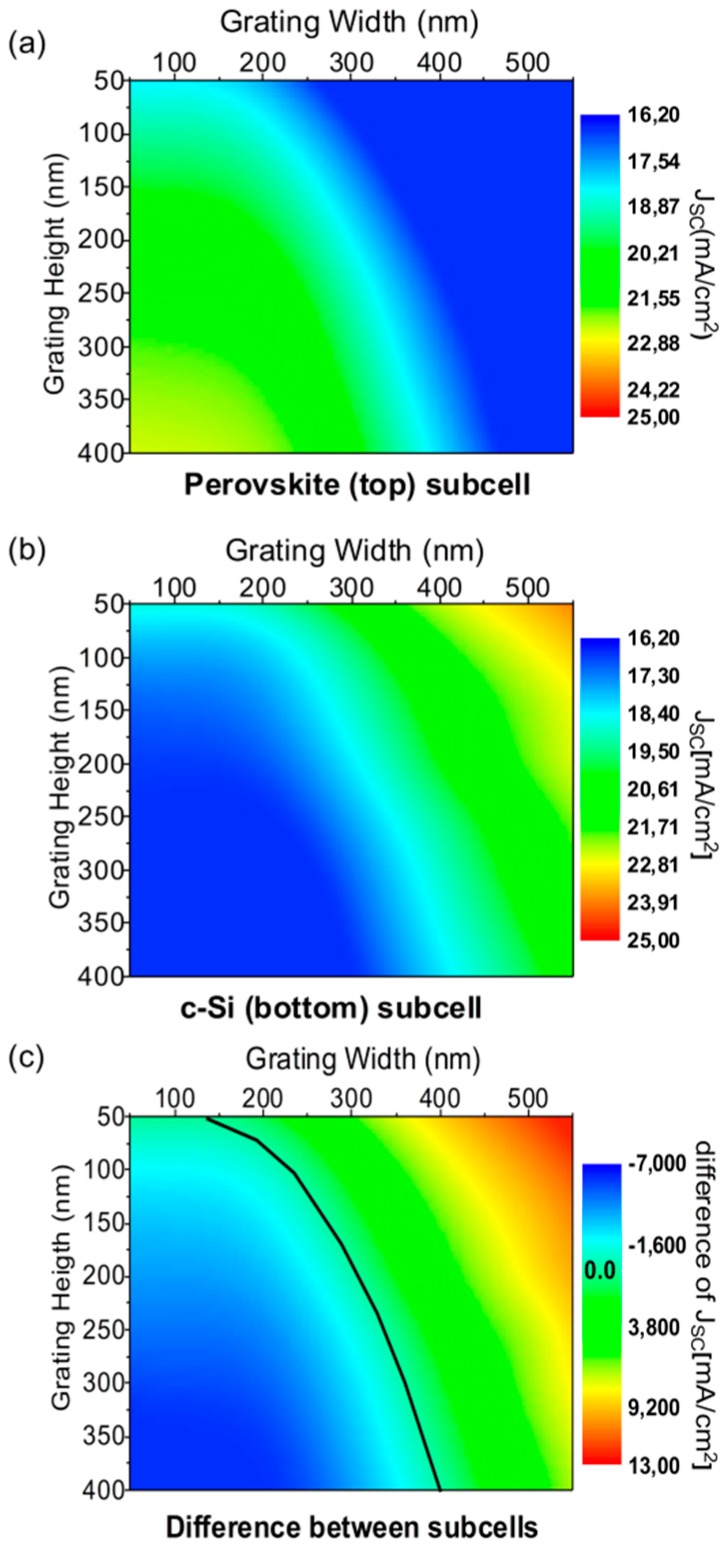
Simulated values of the short-circuit current density (*J_SC_*) in the perovskite (**a**) and the c-Si (**b**) subcells as a function of the geometrical parameters (width and height) of the proposed grating. (**c**) Difference of the generated currents in each subcell as a function of the geometrical parameters of the grating. The values producing the perfect current matching (null difference) are highlighted with a black curve.

One of the most important challenges in the design of tandem solar cells is to achieve an efficient absorption in both active layers in their corresponding bandgap ranges. This requires an efficient transmission of the non-absorbed wavelengths from the top to the bottom layer. In this work, we propose to structure the perovskite layer as a one-dimensional grating instead of a planar slab, acting as a metasurface. Figure 1a shows the complete stack of the different layers. This figure only shows those relevant for the optical behavior, for the sake of simplicity. One of the possible configurations of tandem Perovskite/cSi solar cells is the following one: back contact \ITO (Indium Tin Oxide) \ naSi-H (n-type amorphous Silicon Hydroginated) \ iaSi-H (intrinsic type) \n-c-Si (n-type crystalline Silicon) \ iaSi-H \ paSi-H (p-type amorphous Silicon Hydroginated) \ ITO \ ETL \ Perovskite \ HTL \ top contact. The pin aSi-H layers are a requirement to generate a suitable electric field to separate the solar photo-generated charge carriers in the c-Si active layer. ETL and HTL are the electron and hole transport layers that are required to extract carriers from the Perovskite active layer. However, the thickness of the pin aSi-H is too small [25] and has negligible optical losses. Moreover, we previously simulated the complete stack to check this. So hereinafter, we exclude them from our calculations. Figure 1b,c depict the detail of the materials of the different considered layers and a scheme of the perovskite subcell with a description of the different geometrical parameters. We define the dimensions of the dielectric grating (*GW* and *GH* in Figure 2c) instead of the perovskite ones, because they control the dimensions of the whole metasurface, including perovskite.

The layer structure of the device (from top to bottom) is: antireflection coating (MgF_2_, 105 nm)/transparent conductive oxide (IZO, 44 nm)/hole transport layer (Spiro-OMeTAD, *T_HTLl_* = 10 nm, *T_HTL2_* = 160 nm)/Perovskite (MAPbI_3_, *T_HTL2_*+ *GH* + *T_ETL2_*)/Dielectric layer (Si_3_N_4_, *GH*)/electron transport layer (TiO_2_, *T_ETL2_* = 30 nm, *T_ETL1_* = 10 nm)/transparent conductive oxide (ITO, 44 nm)/crystalline Silicon c-Si (200 μm)/Silver (300 nm). These values and materials were initially considered like those of the planar structure in reference [25] to properly validate our model by taking this planar cell as a reference as it is described in the Supporting information. Although the perovskite materials are cheap and the diffusion length of the optically generated carriers is long (around 1 μm), the perovskite thickness in such a tandem device must be limited to that value producing the exact current matching with the one produced in the c-Si layer. We show it in the Supporting Information (Figure S1). Besides the previous description, some geometrical parameters were changed to optimize the performance of the proposed device, as we explain below. The complex refractive indices of the materials were obtained from [26,27,28,29,30,31,32,33,34].

By introducing the 1D perovskite grating, we try to optimize the solar cell performance by:

1. Efficiently guiding long-wavelength photons to the silicon layer in order to increase light absorption in this subcell.
2. Producing strong diffuse scattering at short wavelengths to increase the absorption in the perovskite.
3. Tuning the *J_SC_* in both cells through the geometrical parameters of the grating to achieve the best current matching.
4. Increasing the interface surface between the perovskite and the charge transport materials (HTL and ETL), to enhance the charge collection of the photogenerated carriers. Additionally, it reduces the perovskite transport layers interfacial problems that appear with large scale plane surfaces [35].
5. Reducing the parasitic losses.

We computed optical absorption in each layer and the generated short-circuit current density using the finite element method (FEM), as described in the Supporting Information. The optimization of the proposed device was carried out by optimizing the geometrical properties of the grating. We remark that our optimization study analyzed the geometrical properties of the grating in the dielectric layer, in both height and width (*GH* and *GW* in Figure 1c, respectively). Thus, the thickness of the perovskite layer was also varied because it was the sum of *GW*, T_HTL2_ and T_ETL2_ (T_perovskite_ = *GW* + T_HTL2_ + T_ETL2_). The width of the unit cell (dashed line in Figure 1) was arbitrarily fixed to 1 μm. So, if the grating width increased, the width of the perovskite decreased (*GW* + perovskite width = 1 μm).

During the optimization process, we also considered a common way to enhance the performance of solar cells: adding a textured back contact. This technique allows an increment of the diffuse scattering at the last interface, increasing the diffuse reflection of photons into the silicon subcell and then the probability to be absorbed. In this sense, we explored different profiles of this textured interface. The results of these optimization processes are discussed in the next Section.

## 3. Results and Discussion

In this section we demonstrate that the proposed metasurface improves solar cell optical performance. In particular, we show that it produced an efficient light management, resulting in an improvement in both absorption and *J_SC_*, keeping a perfect current matching. We also carried out a further enhancement of the device by introducing a textured back contact. All these results are compared with the reference planar structure [25], as stated in the Supporting Information (Figure S1).

The first step was the optimization of the geometry to maximize the short-circuit current density (*J_SC_*) while maintaining the required current matching. Sweeping *GW* and *GH*, we computed *J_SC_* in both the perovskite and c-Si subcells, as shown in Figure 2a,b, respectively.

As was previously commented, optimum performance involves the maximum current value maintaining the current matching between the subcells. To this purpose, Figure 2c plots the difference between *J_SC_* in both subcells. The black line marks the null difference, i.e., where the current matching is achieved. Although there was a set of *GW-GH* pairs producing the current matching, the maximum current value (*J_SC_* = 18.8 mA/cm^2^) was produced at *GW* = 300 nm and *GH* = 225 nm. This was an improvement of a 16% respect to the planar cell (see Equation 3 in the Supporting information for an exact definition of the enhancement factor in %). These optimized geometrical parameters involved a 12% increment of the total amount of perovskite compared to the reference planar cell. An increment of the amount of perovskite directly meant an increment of the absorption in the corresponding wavelength range. However, we checked that a planar cell with the same volume of perovskite would only produce a *J_SC_* = 16.7 mA/cm^2^. Even more, this was not a realistic case, because there would not be current matching with the c-Si subcell. Consequently, there must be other physical effects producing the obtained improvement.

**Figure 3:**
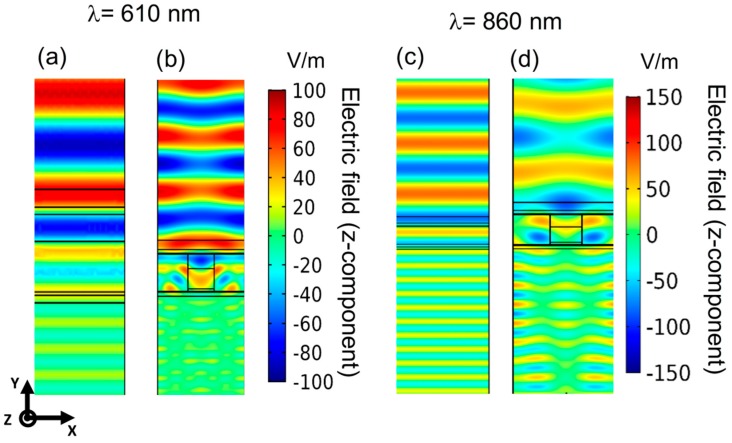
Simulation of the z-component of the total electric field in a monolithic perovskite-silicon tandem solar cell under normal incidence and with a TE polarization considering a planar, (**a**,**c**), and the proposed nanostructured configuration, (**b**,**d**). While (**a**,**b**) uses an incident wavelength in the perovskite absorption range (610 nm), (**c**,**d**) are calculated in the silicon absorption range (860 nm).

To figure out the physical effects involved in the structure, Figure 3 depicts the z-component of the electric field inside both a planar (Figure 3a,c) and the proposed structured solar cell (Figure 3b,d) at wavelengths of 610 nm and 860 nm. The incident beam had a TE (transverse electric) polarization and normal incidence. Similar results considering a TM (transverse magnetic) polarization and 50° incidence angle, reproducing an extreme solar position along the day, are shown in the Supporting Information (Figure S2).

On the one hand, 610 nm is into the high-reflectance band for the c-Si cell, and inside the absorption band of the perovskite. While Figure 3a shows the typical propagation through a planar structure, with absorptions into each one of the layers, in the structured cell (Figure 3b) a remarkably different field distribution appears (note that the color scale is the same in both planar and nanostructured graphs): an electric field concentration appears in the perovskite volume, increasing the absorption rate according to Equation 1 of the Supporting Information. This spatial field profile may be produced by the scattering effects of the nanostructured geometry in the metasurface. On the other hand, 860 nm is a wavelength in the c-Si absorption range. In this case, a diffractive lens effect, due to the grating metasurface, arose in the c-Si subcell (Figure 3d), showing an efficient guiding of light inside it in respect to the planar one (Figure 3c).

These effects were almost independent on both the incident light direction and the polarization, as they were also produced without significant variations changing these conditions (see Figure S2 of the Supporting Information).

**Figure 4:**
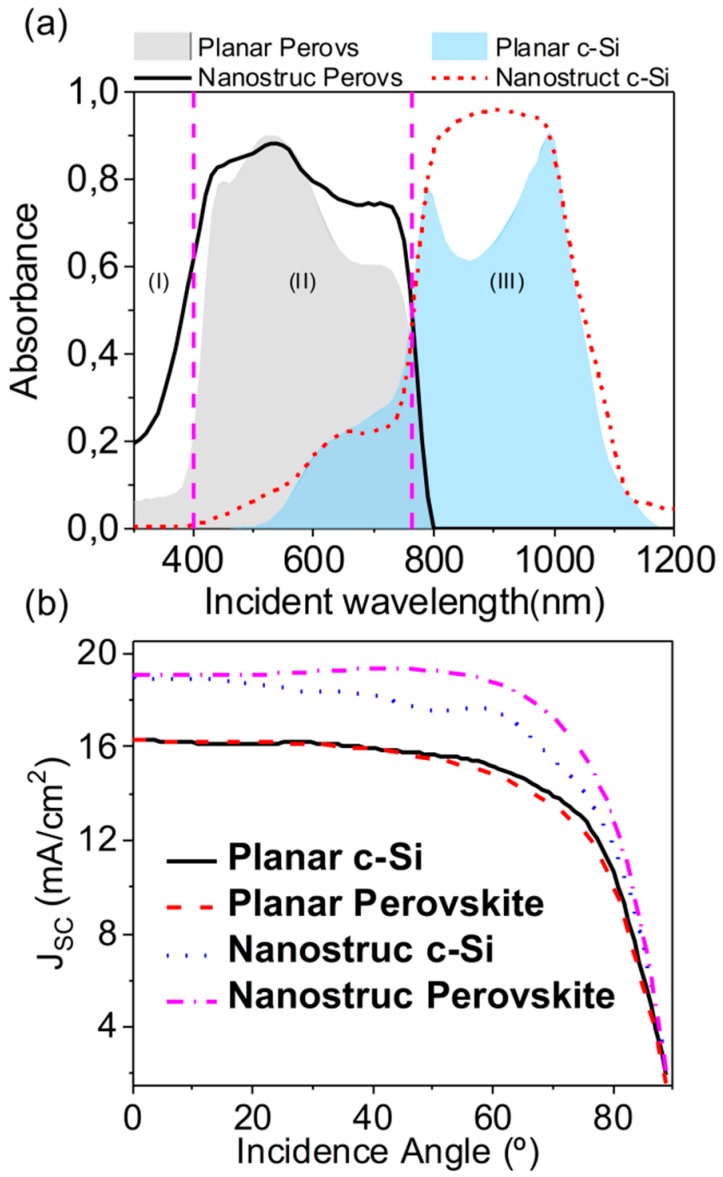
(**a**) Comparison of the spectral evolution of the absorbance in each subcell between a typical monolithic tandem solar cell with a planar perovskite (gray area) and crystalline silicon (blue area) layers and that of the proposed optimum structure with a nanostructured perovskite (black solid line) and c-Si (red dashed line) layers along the solar spectrum. TE polarization is considered. Vertical dashed lines delimit three interesting spectral regions (see explanation in the discussion). (**b**) Short-circuit current density generated in each subcell of a planar device (c-Si subcell, solid black line; perovskite subcell, dashed red line) and the proposed device (c-Si subcell, dotted blue line; perovskite subcell, dash-dotted pink line) as a function of incidence angle of the incoming light.

Figure 4a shows the simulated total effective absorption (adding the ones at c-Si and perovskite). For the sake of comparison, we also plotted the planar structure result, in both perovskite and c-Si layers. As before, only TE results appeared. TM results can be seen in the Supporting Information (Figure S3). An evident enhancement in both subcells was observed. The increment in the c-Si band (800–1000 nm, region III in Figure 4a) was due to the increment of light reaching this layer as a result of the guiding ability of the new top subcell, as Figure 3d revealed. At shorter wavelengths, in the perovskite absorption band, (region II in Figure 4a), the spatial concentration of light inside the grating (Figure 3b) allowed a higher absorption, which was proportional to the square of the electric field (see Equation 1 of Supporting Information). At even shorter wavelengths (region I in Figure 4a) the absorption increment was a consequence of the decrement of the reflectance due to the proposed structure.

An efficient and realistic proposal should hold this enhancement at different incidence angles, following the Sun directions. Figure 4b shows the angular dependence of the simulated *J_SC_* in the active regions for both the planar and the nanostructured geometries. The geometrical parameters of the nanostructure were the previous optimum ones (*GH* = 225 nm, *GW* = 300 nm, producing *J_SC_* = 18.8 mA/cm^2^ at normal incidence). Following the results of Figure 4a, the enhancement of the absorption led to higher values of the photocurrent at every incidence angle. The nanostructured configuration losses were the perfect matching condition from 20°, as they were optimized in 1D and thus were slightly sensitive to polarization. Despite that fact, an average increment of 15% in the enhancement factor respect to the planar cell was achieved up to 70°. On the other hand, a deeper study of the involved materials would be interesting to check the possibilities of manufacturing this device, and will be analyzed in further research.

Textured surfaces were checked in many previous reports [36,37], intending to achieve a further enhancement of the optical absorption in the active layer by increasing the optical path in it. So far, we have established the effect of the nanostructured perovskite layer in the tandem solar cell. The next step of our analysis was checking the compatibility of inserting one of these textured back contacts with the nanostructured top cell, and especially checking if their geometrical misalignment was a drawback for the effect that we had achieved up to now.

**Figure 5:**
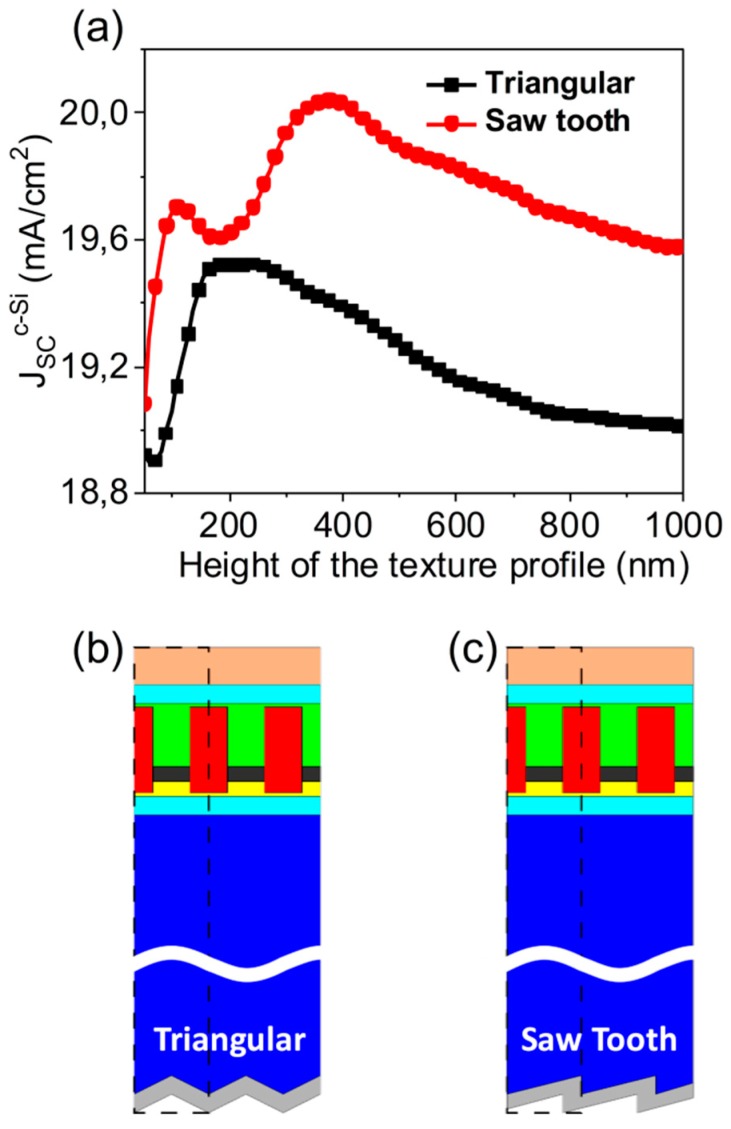
(**a**) Short-circuit current density generated in the c-Si subcell of the proposed device considering a textured back contact with a triangular (black squares) or a saw tooth (red circles) profile, as a function of the height of the profile. Schemes of the device with the considered profiles for the back contact are included in (**b**,**c**), respectively.

Front and back-textures led to further improvement in the *J_SC_*, by increasing the light diffusion into the c-Si layer. In our proposal, the upper contact already included an antireflection coating. For this reason, we focused on analyzing the influence of texturing the back contact of the device on its performance. In particular, we explored two typical profiles for the Ag back contact: a triangular and a saw tooth (Figure 5b,c, respectively). Then, we repeated the analysis to obtain the maximum *J_SC_* at at the c-Si subcell using these textured contacts, varying the height of the profile as the optimization parameter (Figure 5a).

As these textured profiles provided an enhancement of the short-circuit current density only in the c-Si subcell, they perturb the current matching condition. For this reason, the geometrical parameters of the metasurface must change again to recover this condition. This confirms the ability of the proposed metasurface to optimize the solar cell performance by tuning its geometry. After the optimization process, we obtained a net matching current of 19.35 mA/cm^2^ (*GW* = 300 nm and *GH* = 220 nm) for the triangular case, and 19.55 mA/cm^2^ (*GW* = 300 nm and *GH* = 230 nm) for the saw tooth case. As can be seen, the saw tooth profile involved an increment of the 20.5% respect to the planar one (the enhancement factor of the triangular one is 19.29%).

Another detail to take into account is the alignment between the textured contact and the proposed metasurface. We have also studied the geometrical misalignment between the back-contact profile and the metasurface, and no significant difference has appeared (the greatest *J_SC_* differences being ~0.2 mA/cm^2^), showing that the alignment of both structures is uncorrelated. This reinforces the idea that this back-contact texturing only provides a better diffuse reflection and light redirection towards the c-Si active layer, without any remarkable influence on the top subcell.

**Figure 6:**
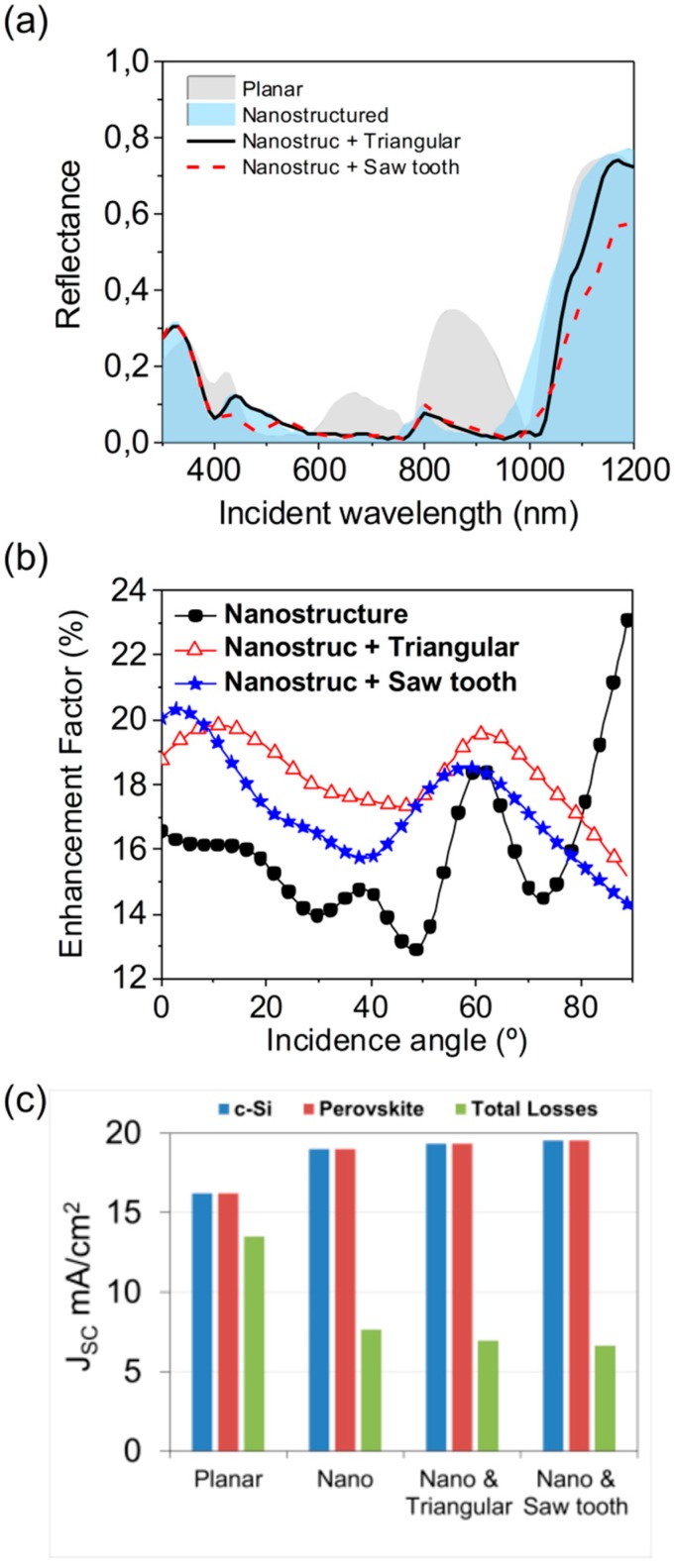
(**a**) Total reflectance of the device, within the solar spectrum, under the four different configurations: planar (gray area), nanostructured (blue area), nanostructured with a textured back contact with a triangular (black solid line) or a saw tooth (red dashed line) profile. (**b**) Enhancement factor of the short-circuit current density of the three proposed configurations (nanostructured, black solid circles; nanostructured + triangular back contact, red hollow triangles; nanostructured + saw tooth back contact, blue stars) with respect to the typical planar one as a function of the angle of incidence. (**c**) Comparison of the effective absorbance in the active layers (c-Si, blue; perovskite, red) and the parasitic one or total losses (green).

Figure 6a shows the reflectance of the device in the solar spectrum for every considered configuration: planar reference, nanostructured, and nanostructured with textured back contacts (triangular and saw tooth). In the efficient absorption range of the device (from 400 nm to 1100 nm as seen in Figure 4 and Figure S1), the nanostructured configuration offered a remarkable reduction of the reflectance, especially between 600 and 1000 nm. Additionally, the texturization of the back contact allowed for reducing the reflectance even more. Particularly, the saw tooth profile produced negligible values of the reflectance between 440 nm and 570 nm, while in the rest of the range triangular and saw tooth offered similar values. 

Besides this, the response of the device under an oblique incidence improved when the back contact was textured. Figure 6b depicts the enhancement factor (*EF*, see definition at the Supplementary Information) of *J_SC_* for each one of our proposals in respect to the planar one as a function of the incident angle. From a general point of view, a remarkable enhancement (over 13%) was obtained for all the angles with all our proposals. The triangular profile got a higher enhancement in a wide angular range, but the saw tooth profile was the best solution for the usual operation of a solar cell (close to a normal incidence during the most part of the day, with values of *EF* over 20%).

In the Supporting information (Figure S4), we show the detailed absorbance of every configuration. 

Finally, Figure 6c plots the obtained *J_SC_* in both active layers (perovskite and c-Si) and in the other layers of the device, which are considered as parasitic losses. As can be seen, the inclusion of the nanostructure improved not only the perovskite subcell current generation but also the c-Si one respect to the planar one. At the same time, the parasitic losses decreased. We guess that this reduction was mainly produced by:

- The strong light confinement in the perovskite volume (see Figure 3). For instance, it produced 62% lower parasitic losses in the HTL in respect to the planar cell.
- The reduction of the total reflectivity (as seen in Figure 6a), decreasing a 48% respect to the planar cell for the nanostructured proposal and reaching down to 66% when texturing the back contact as well. Even in this matter, the saw tooth profile was better than the triangular one.

## 4. Conclusions

In summary, we have proposed a novel design to manage light in a perovskite –silicon tandem solar cell by transforming the perovskite layer into a hybrid metasurface. Actually, this metasurface was produced by embedding perovskite blocks into a nanostructure formed by the transport layers and a dielectric spacer. We have identified two important effects to improve the solar cell optical performance by this nanostructured grating: on the one hand, the enhancement of light confinement into the perovskite volume through resonant scattering behaviors; on the other hand, an efficient light guiding to the bottom part of the device at wavelengths corresponding to the silicon absorption range. These effects produced a reduction of reflections and then smaller optical losses than in a planar case. Additionally, we increased the contact surface between the transport layers (HTL and ETL) and the perovskite, so we guess that an improvement of the charge extraction may also be observed.

We optimized the geometrical properties of the metasurface to achieve a maximum matched short-circuit current density. Besides the detailed values, which may be changed considering other configurations, the proposed metasurface was able to provide an enhancement of the short-circuit current density of 16.83%. This value was also improved by including a textured back-contact. Several state-of-the-art works use random texture because it is easier and cheaper than some periodic structures. However, periodic profiles provide a better performance and reproducibility. The development of low-cost and large-scale nanofabrication techniques (e.g., nano-imprint, photolithography) allow for the use of these structures in real systems. We observed that a saw tooth feature provided better device performance than a typical triangular one, producing an enhancement factor of a 20.5% respect to the planar case.

Although we propose a 1D metasurface, which is slightly polarization dependent, we have shown that the obtained improvements in the active layers work for both orthogonal linear polarizations and every incident light angle. Further research will be done for determining the influence of the polarization in the losses of the non-active layers. 

We conclude that the findings in this paper can be used to fabricate a perovskite/c-Si tandem solar cell with improved efficiency, providing to the manufacturers a wide set of alternatives in terms of the geometrical dimensions to be used in the device.

## Supplementary Materials

The following are available online at https://www.mdpi.com/2079-4991/9/5/791/s1, Figure S1: (**a**) Spectral evolution of the absorbance in each subcell of a typical monolithic tandem solar cell composed by a perovskite (gray area) and a crystalline silicon (blue area) layers along the solar spectrum. The total reflectance (black dashed line, right axis) and absorbance (red solid line) of the device are also included. (**b**) Short-circuit current density generated in c-Si (black circles) and perovskite (red triangles) subcells as a function of the thickness of the top active layer (perovskite layer), Figure S2: Simulation of the z-component of the total electric (upper panel) and magnetic field (bottom panel) in a monolithic perovskite-silicon tandem solar cell at an incident wavelength of 860 nm. Calculations for a planar perovskite layer under normal and a non-normal incidence (50°) are shown in (**a**), (**e**) and (**c**), (**g**) respectively. The proposed solar cell with a nanostructured perovskite metasurface is shown in (**b**), (**f**) and (**d**), (**h**) for normal and non-normal incidence, respectively. Incident beam is linearly polarized with a transverse magnetic (TM) configuration, Figure S3. Comparison of the spectral evolution of the absorbance in each subcell between a typical monolithic tandem solar cell with a planar perovskite (gray area) and a crystalline silicon (blue area) layers and that of the proposed optimum structure with a nanostructured perovskite (black line) and a c-Silicon (red line) layer along the solar spectrum. An incident TM polarization is considered, Figure S4. Comparison of the spectral evolution of the absorbance in each subcell of a monolithic tandem solar cell considering the proposed nanostructured perovskite region and three different back-contact configurations: a planar one (filled areas), a triangular profile (dashed lines) and a saw tooth one (solid lines). Both linear polarizations, TE (**a**) and TM (**b**), are considered.
