# Supplementary material for: Efficient Light Management in a Monolithic Tandem Perovskite/Silicon Solar Cell by Using a Hybrid Metasurface
Source: Nanomaterials (Basel). 2019 May 23;9(5):791. doi: 10.3390/nano9050791 (PMC6566752; doi:10.3390/nano9050791)
Supplement: Supplementary file 1 [file nanomaterials-09-00791-s001.pdf]

Supporting Information

Efficient Light Management in a Monolithic Tandem Perovskite/Silicon Solar Cell by Using a Hybrid Metasurface

Mahmoud H. Elshorbagy [1,2], Braulio García-Cámara [1,*], Eduardo López-Fraguas [1] and Ricardo Vergaz [1]

[1] GDAF-UC3M, Departamento de Tecnología Electrónica, Universidad Carlos III de Madrid. Avda. Universidad, 30. Leganés, Madrid E28911, Spain

[2] Grupo Complutense de Óptica Aplicada, Departamento de Óptica, Facultad de Óptica y Optometría, Universidad Complutense de Madrid, Madrid E28037, Spain

* Correspondence: brgarcia@ing.uc3m.es; Tel.: +34-916-248-869

Theoretical Considerations

In order to characterize the proposed device, we calculated the light absorption in each region. In this sense, the absorbed power density at a certain incident frequency (or wavelength) could be calculated from the divergence of the Poynting vector as [1]:

$$P(\omega) = \frac{1}{2}\,\omega\varepsilon''(\omega)|E(\omega)|^2 \tag{1}$$

where ω is the angular frequency of the incoming radiation, ε'' is the imaginary part of the dielectric permittivity of the material, and $|E(\omega)|^2$ is the intensity of the electric field inside each material. This means that a strong confinement of the electric field in a certain area produces higher absorption in that specific region. While light absorption in the active layers contributes to the photocurrent generation, there is also absorption in the other layers, which is considered as a loss. Despite the fact that we desire to enhance the efficient absorption in the active layers, it is also important to take into account the parasitic ones to avoid potential thermal effects. The absorption rate is the absorbed power density over the total incident power. The volume integration of this absorption rate in the active layers gives us the total effective absorbance of the device, $A(\lambda)$. This can be also numerically obtained through reflectance and transmittance calculations.

From an electrical point of view, the short-circuit current density (J_{SC}) reflects the response of the device and it is an indirect measurement of its quantum efficiency. In fact, we chose this parameter to estimate the quantitative improvement of our proposal. Assuming that each absorbed photon creates an electron-hole pair contributing to the short-circuit current density, J_{SC} can be given over the solar spectrum, as follows in terms of the wavelength (λ) [2]:

$$J_{SC} = \int q\,\frac{\lambda}{hc}A(\lambda)\phi_{AM1.5G}(\lambda)d\lambda \tag{2}$$

where$q$ is the electron charge, c is the speed of light in vacuum, h is the Planck's constant, and $\varphi_{AM1.5G}$ (λ) is the standard solar spectral irradiance AM1.5G [3].

Numerical model

We used the Finite Element Method (FEM) to simulate the proposed structure and to obtain the previous parameters. We used the commercial tool COMSOL Multiphysics ©. As it is well known, this method solves the Maxwell equations by discretizing the structure into small elements and solving the fields in each element. As a tandem solar cell involves different layers with different thickness, both in nanometric and micrometric scales, the choice of the mesh is crucial. In this sense, several simulations were performed to optimize our numerical model. We implemented a 2D model

assuming a normal illumination with an incident plane wave under both TE and TM polarizations and wavelengths in the range of the solar spectrum (300 nm–1200 nm). The solar irradiance standard, AM1.5G [3], was implemented as the input power of our model. This means that the global incident irradiance is 1000 W/m^2. Using the unit cell depicted in Figure 1 in the article, periodic boundary conditions were set on the lateral boundaries, while top and bottom boundaries of the computational domain included perfect matched layers (PML) with absorbing boundary conditions. The absorbed power at each layer was calculated for the internal COMSOL power losses function, and the total reflectance and transmittance were retrieved from the S parameters.

We used a previous study [4] to verify our model and to use it as a reference in order to show the improvements achieved. Its geometrical parameters were the starting point for our calculations. The layer structure of the device (from top to bottom) was: antireflection coating (MgF_2, 105 nm) / transparent conductive oxide (IZO, 44 nm)/ hole-transport layer (Spiro-OMeTAD, 160 nm) / perovskite ($MAPbI_3$, 260 nm) / electron-transport layer (TiO_2, 30 nm) / transparent conductive oxide (ITO, 44 nm) / crystalline Silicon c-Si (200 µm) / Silver (300 nm). Figure S1a shows both the absorptance and reflectance of this reference planar device obtained through our model. A simple comparison shows an accurate reproduction of the results of this previous work. This figure also shows that these kinds of tandem solar cells were conceived to obtain a joint effect in the absorption. While perovskite area acts in the low wavelength range (from 400 nm to 800 nm), the c-Si subcell mainly absorbs from 700 nm to 1100 nm. Unfortunately, the total reflectance dramatically increases due to the effect of the multilayer interfaces. For instance, absorption decays by this effect around 680 nm (reaching 85%) and 860 nm (reaching only a 65%), as can be seen in Figure S1a. For this reason, these multilayer solar cells require solutions to minimize this reflectivity and to get an optimum light coupling between both active layers.

In contrast to 4-terminal configurations in which each subcell is independent and they can separately operate in its optimum performance, monolithic tandem solar cells require a trade-off between the generated photo-currents in each subcell [5,6] to satisfy the Kirchoff's law for series connections. In this sense, we firstly used our COMSOL model considering a planar perovskite layer, our reference planar structure henceforth, to determine the thickness of the perovskite planar layer that offers a perfect matching of the photocurrent between both subcells (c-Si and perovskite) of the tandem cell. The control of the thin film perovskite layer to obtain the perfect matching was easier and more cost-effective than that of the silicon wafer. Figure S1b shows J_{SC} in both active layers as a function of the perovskite thickness. The thicker the perovskite layer, the larger the probability to generate carriers. In contrast, this also reduces the quantity of photons reaching the c-Si subcell, which photo-current decreases (Figure S1b). The thinner the perovskite layer, the lower its absorption capabilities. The best match was obtained in the intersection between the plotted current densities, occurring at a thickness of 260 nm. At this point the short-circuit current density was 16.22 mA/cm^2 in both subcells. This J_{SC} will be our reference value to be improved hereafter. Actually, to evaluate the improvement of the proposed device, we defined the enhancement factor as follows:

$$EF = \frac{J_{SC_{proposed}} - J_{SC_{planar}}}{J_{SC_{planar}}} \times 100(\%) \qquad (3)$$

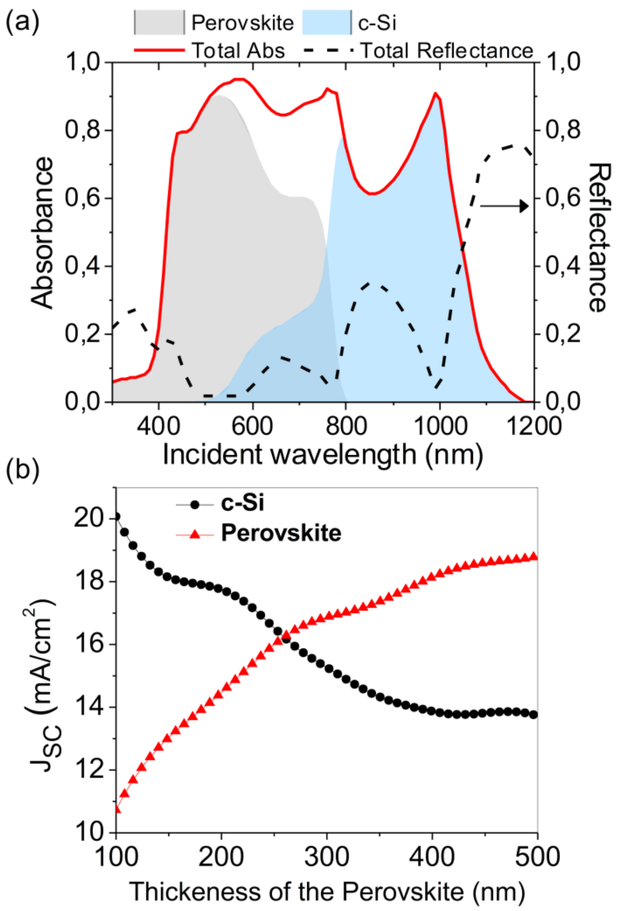

Figure S1. (**a**) Spectral evolution of the absorbance in each subcell of a typical monolithic tandem solar cell composed by a perovskite (gray area) and a crystalline silicon (blue area) layers along the solar spectrum. The total reflectance (black dashed line, right axis) and absorbance (red solid line) of the device are also included. (**b**) Short-circuit current density generated in c-Si (black circles) and perovskite (red triangles) subcells as a function of the thickness of the top active layer (perovskite layer).

Light Management Comparison

Figure S2 shows the spatial distribution of the electric (upper panel) and the magnetic (lower panel) fields inside a planar and a nanostructured perovskite-silicon tandem solar cell under a normal and non-normal incidence. The incident wavelength was 860 nm. The desired effects of light confinement in the perovskite and light guiding into the c-Si subcell were clearly observed in all the cases. This shows the utility of the proposed device under real operation conditions.

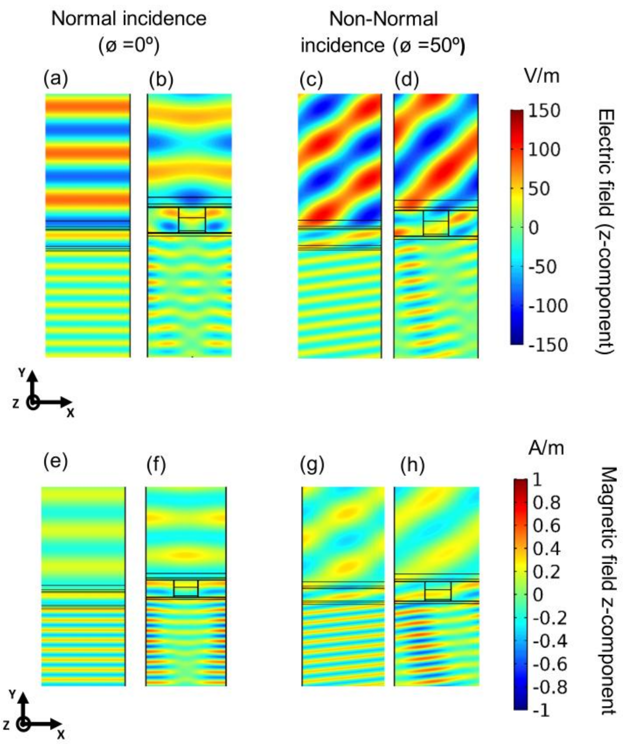

Figure S2. Simulation of the z-component of the total electric (upper panel) and magnetic field (bottom panel) in a monolithic perovskite-silicon tandem solar cell at an incident wavelength of 860 nm. Calculations for a planar perovskite layer under normal and a non-normal incidence (50°) are shown in (**a**), (**e**) and (**c**), (**g**) respectively. The proposed solar cell with a nanostructured perovskite metasurface is shown in (**b**), (**f**) and (**d**), (**h**) for normal and non-normal incidence, respectively. Incident beam is linearly polarized with a transverse magnetic (TM) configuration.

Solar cell Performance under TM Polarization

The absorbance spectra of the planar and the proposed devices appear in Figure S3. In this case, the incident beam was linearly polarized with a transverse magnetic (TM) configuration. There were no significant changes respect to the TE polarization. Although the proposed metasurface is 1D, we observed that the global operation of the device was almost independent on the incident polarization.

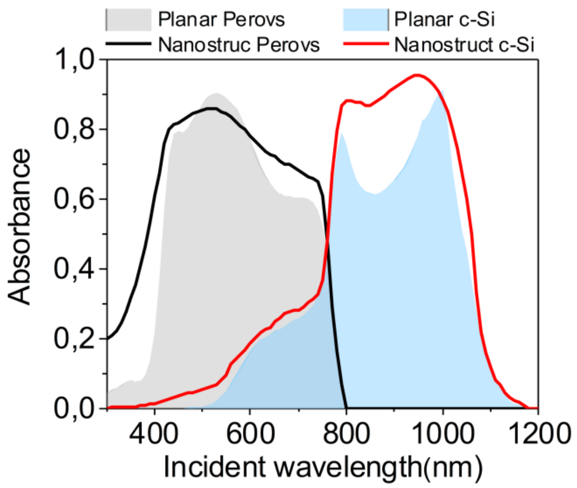

Figure S3. Comparison of the spectral evolution of the absorbance in each subcell between a typical monolithic tandem solar cell with a planar perovskite (gray area) and a crystalline silicon (blue area) layers and that of the proposed optimum structure with a nanostructured perovskite (black line) and a c-Silicon (red line) layer along the solar spectrum. An incident TM polarization is considered.

Comparison of the Absorbance Spectra with Textured Contacts

Figure S4 shows a comparison of the absorbance spectra of both textured and non-textured back contact proposed solar cell. Both TE and TM incident polarizations were considered. Although the difference was not significant, a remarkable expansion of the effective spectral range at long wavelengths was observed in the TE polarization when using textured back contacts. Additionally, both considered profiles provide similar results, although the saw tooth one gives a numerically better performance.

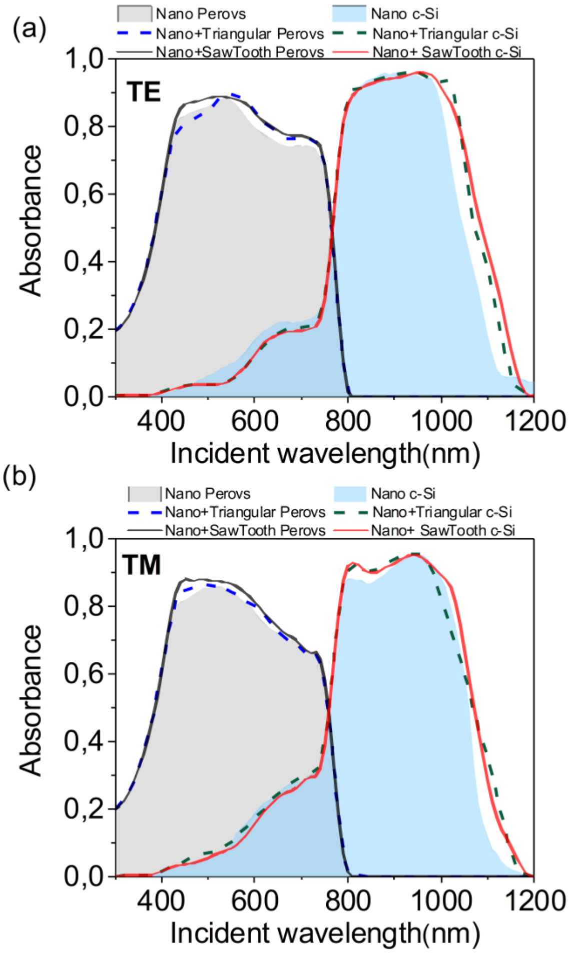

Figure S4. Comparison of the spectral evolution of the absorbance in each subcell of a monolithic tandem solar cell considering the proposed nanostructured perovskite region and three different back-contact configurations: a planar one (filled areas), a triangular profile (dashed lines) and a saw tooth one (solid lines). Both linear polarizations, TE (**a**) and TM (**b**), are considered.

References

1. Boroumand, J.; Das, S.; Vázquez-Guardado, A.; Franklin, D.; Chanda, D. Unified electromagnetic-electronic design of light trapping silicon solar cells. *Sci. Rep.* **2016**, *6*, 31013.
2. Elshorbagy, M.H.; Alda, J. Funneling and guiding effects in ultrathin aSi-H solar cells using one-dimensional dielectric subwavelength gratings. *J. Photon. Energy* **2017**, *7*, 017002.
3. National Renewable Energy Laboratory (NREL). MS Excel™ spreadsheet file. Available online: https://rredc.nrel.gov/solar/spectra/am1.5 (accessed on 20 May 2019).
4. Grant, D.T.; Catchpole, K.R.; Weber, K.J.; White, T.P. Design guidelines for perovskite/silicon 2-terminal tandem solar cells: An optical study. *Opt. Express* **2016**, *24*, A1454–A1470.
5. Jäger, K.; Korte, L.; Rech, B.; Albretch, S. Numerical optical optimization of monolithic planar perovskite-silicon tandem solar cells with regular and inverted device architectures. *Opt. Express* **2017**, 25, A473–A481.
6. Iftique, S.M.; Jung, J.; Yi, J. Improved efficiency of perovkite-silicon tandem solar cell near the matched optical absorption between the subcells. *J. Phys. D* **2017**, *50*, 405501.
